# Approach to Thyroid Nodules: Diagnosis and Treatment

**DOI:** 10.7759/cureus.52232

**Published:** 2024-01-13

**Authors:** Aram H AlSaedi, Dalia S Almalki, Reem M ElKady

**Affiliations:** 1 College of Medicine, Taibah University, Madinah, SAU; 2 College of Medicine, Taibah University, Jeddah, SAU; 3 Radiology and Medical Imaging, Taibah University, Al-Madenah, SAU

**Keywords:** ultrasonography, tsh, nodules, malignant, fna, bethesda

## Abstract

Thyroid nodules (TNs) are prevalent and found in up to 50% of individuals. While most TNs are benign, some can be malignant. The evaluation of TNs is crucial to rule out malignancy and identify those requiring surgical intervention. This study aimed to clarify the reported prevalence of TNs, focusing specifically on their various types, assessment and diagnostic processes, current evaluation methods, and evidence-based management. It also provides recommendations for follow-up. TNs are typically found during physical exams or incidentally during imaging procedures. Routine laboratory and clinical evaluations of TNs are common. Ultrasound is the preferred imaging method to determine if a TN needs a biopsy. Fine-needle aspiration (FNA) is crucial in deciding whether surgery or surveillance is necessary. TNs that show suspicious features on the ultrasound may require cytologic analysis to assess the risk of malignancy. The effectiveness of several supplementary molecular tests is still uncertain, although some studies report promising results. The management and treatment approach for TNs primarily depends on the results of FNA cytology and ultrasound characteristics. The optimal treatment strategy for TNs ranges from straightforward follow-ups for low-risk cases to surgical intervention for high-risk patients. Rather than adopting a uniform approach, clinicians should assess each patient on a case-by-case basis using current knowledge and a collaborative, multidisciplinary method.

## Introduction and background

Introduction

Thyroid nodules (TNs) are commonly found in clinical practice. The American Thyroid Association defines a TN as a "discrete lesion in the thyroid gland that is distinct radiologically from the surrounding thyroid parenchyma" [1]. It is common for individuals in the general population to exhibit irregular thyroid functions, which can adversely affect their health. Consequently, it is crucial to have a range of economical tests available for the precise diagnosis and specific treatment of TNs to prevent potential complications.

Screening methods and population demographics influence the prevalence of TNs. Factors such as advancing age, being female, having an iron deficiency, and a history of thyroid radiation [2] can increase the risk. A physical examination alone may reveal a 5-7% prevalence. However, ultrasound screenings show a higher prevalence of 20-76%, which aligns with autopsy findings [3]. Notably, TNs occur roughly four times more frequently in women compared to men [4].

The most frequently observed cases of TNs include colloid nodules, Hashimoto's thyroiditis, subacute thyroiditis, cysts, follicular adenomas, and thyroid cancer. Evaluating TNs clinically is crucial for assessing thyroid function, determining the extent and potential risk of mass effect, and ruling out thyroid cancer. However, the primary difficulty clinicians face in managing patients with TNs lies in accurately identifying the small percentage of patients with thyroid cancer who would benefit from surgical intervention and additional therapy. This must be achieved in a way that minimally impacts patients with benign TNs [5].

The initial clinical evaluation of the thyroid gland for an accurate diagnosis and appropriate treatment strategy requires gathering the patient's history, conducting a physical examination, and interpreting imaging and lab test results. Adhering to established guidelines for these basic steps is essential to avoid exposing the patient to unnecessary tests and prevent the healthcare system from incurring needless expenses [5].

During the initial evaluation of suspicious TNs, a thorough patient history should be considered, especially those risk factors that suggest the possibility of malignancy [6,7]. The physical examination also plays a critical role in evaluating TNs. This examination should primarily assess the volume, consistency, and characteristics of the nodules. Further, a thyroid ultrasound is recommended for all patients with known or suspected TNs. This ultrasound helps confirm the existence of the nodule, identify any additional nodules or cervical lymph nodes, and evaluate suspicious sonographic features [8].

Additionally, various practical lab tests can assess thyroid function. For instance, blood tests, like the thyroid-stimulating hormone (TSH) test, can differentiate a thyrotoxic nodule from a euthyroid nodule [4].

The fine-needle aspiration (FNA) biopsy is considered the "gold standard" method for diagnosing TNs. It is a quick, safe, sensitive, and cost-effective test for evaluating these nodules [9]. Additionally, there are advanced diagnostic methods available, such as diffusion-weighted magnetic resonance imaging (MRI), positron emission tomography/computed tomography (CT), optical coherence tomography, and optical coherence microscopy [10].

Patients with benign TNs typically do not require surgery. If symptoms of hyperthyroidism arise, they can be lessened with anti-thyroid medications. Hypothyroidism is usually treated with thyroid hormone replacement. After thyroidectomy, any remaining healthy thyroid tissue can be eliminated through radioiodine ablation [5].

The treatment approach for TNs with indeterminate cytology varies based on institutional expertise. Some institutions collect a second FNA sample during the initial biopsy for molecular testing. Others prefer to perform a repeat FNA biopsy after 6-12 weeks. It is usually considered fitting to opt for surgery when TNs appear suspicious of malignancy. Additionally, research suggests that injecting a small quantity of alcohol can potentially eliminate a malignant TN [5].

Rationale

TNs are a prevalent disorder globally, posing a significant public health concern. Notably, there has been an increase in the incidence of TNs in recent years. While most studies suggest that early diagnosis aids management, the best approach to TN management remains disputed. Hence, our review aims to determine the various types of TNs and identify the initial assessment processes and diagnostic techniques used in the evaluation of TNs. In addition, it explores the state-of-the-art processes for the diagnosis and management of TNs. Finally, investigate the available treatment and surgical options concerning TNs.

## Review

Epidemiology

The prevalence of measurements can vary greatly, depending significantly on the selected screening method and the population assessed. For example, there tends to be a higher occurrence of TNs in regions with low iodine intake [2]. Physical examinations alone have identified a 5-7% prevalence of TNs in adults. However, using more sensitive imaging techniques such as high-resolution ultrasound, the prevalence is estimated to be 20-76% within the same population. Autopsy studies have also reported a high prevalence of TNs. Although the majority of TNs are benign, around 8-15% have been found to be malignant. As a result, distinguishing accurately between benign and malignant TNs has been a significant focus of research in this field [11].

TNs are rare in children and more common in women than men during adulthood, with a prevalence four times higher in women [4]. A 20-year research study showed that approximately 5.3% of women are affected by TNs, while only 0.8% of men have the same condition [12]. However, men have a higher rate of thyroid cancer, with an incidence roughly double that of women (8% in men compared to 4% in women) [13].

Several risk factors have been linked to the development and higher incidence of TNs. These include advanced age, gender, insufficient or excessive iodine, a family history of thyroid cancer or polyposis (like Gardner's syndrome), a history of radiation therapy to the head or neck, rapid nodule growth, and symptoms of local invasion, such as difficulty swallowing, neck pain, and hoarseness. Moreover, long-term survivors of hematopoietic stem-cell transplants are found to have an elevated risk of secondary thyroid cancer [14].

Types of thyroid nodules

Various pathologies can present as TNs.

Colloid Nodules

Colloid nodules are non-neoplastic benign nodules that occur due to an increase in thyroid volume. This typically involves an expansion of more than 40 mL or twice the customary volume of an adult's thyroid. We can use ultrasound to detect this overgrowth of normal thyroid tissue. Despite their potential for large growth, goiters remain confined to the thyroid gland itself. Colloid nodules, which are non-cancerous, represent 70% of all nodules [15].

Follicular Adenomas

Follicular adenomas of the thyroid are composed of many small, primitive, or fetal-like follicles that hold little or no colloid. Often called microfollicular, these benign nodules form the majority of adenomas. Diagnosing TNs with follicular features can be challenging because they exhibit a wide spectrum of characteristics, ranging from benign to malignant. For instance, among every 100 follicular neoplasms, 15 might be cancerous. Malignant cells generally have a diverse appearance, while benign cells are almost uniform in size and look. However, follicular cancer cells defy this general rule as they resemble benign tumor cells due to their consistent structure. Hence, each follicular adenoma needs to be surgically excised and thoroughly examined to ensure it is not cancerous [15].

Thyroid Cysts

Cysts constitute 15-25% of all TNs. While some cysts, solely filled with fluid, are usually benign, others represent mixed growths consisting of both solid and fluid parts. These complex mixed cysts have a higher cancer risk. Therefore, due to a greater chance of them being mixed growths, large cysts necessitate continuous monitoring and evaluation [15].

Inflammatory Nodules

Inflammatory nodules often form as a result of extended, chronic thyroid gland inflammation, though they can also occur post-pregnancy. Normally, these benign growths are identifiable through ultrasound and typically do not require further evaluation. They can cause pain in some instances but are often painless. Once inflammation subsides, these nodules usually disappear [15].

Malignant Nodules

While most TNs are harmless, some can signify thyroid cancer. These malignant TNs are usually hard on palpation and can grow significantly large (>3 cm). Typically, they present no symptoms, but when they do, the most common one is a noticeable lump in the neck. Various types of thyroid cancers exist. Previous studies report that approximately 80% to 85% are papillary, 10% follicular, 3% medullary, and 1% anaplastic. Most thyroid cancer types respond well to treatment, but some rare forms can behave aggressively [15].

Initial assessment

The first step in assessing TNs clinically involves gathering the patient's full medical history, conducting a physical examination, and carrying out laboratory tests. Once all crucial information has been compiled, a suitable treatment plan can be scheduled.

History and Physical Examination

Acquiring a thorough medical history, with an emphasis on malignancy risk factors in TNs, is crucial in the initial assessment of all TN patients [16]. Key questions must be posed regarding their medical history, particularly for patients presenting with TN and thyroid gland concerns (Table 1). Evaluation should focus on identifying symptoms associated with hypothyroidism or hyperthyroidism. While many individuals are asymptomatic, some may exhibit evidence of disrupted thyroid hormone levels or nerve involvement. Symptoms of hyperthyroidism include nervousness, heat intolerance, diarrhea, muscle weakness, and loss of weight and appetite. Hypothyroidism may result in cold intolerance, constipation, fatigue, and weight gain, which, in children, is primarily caused by the accumulation of myxedematous fluid. Prompt investigation is warranted when signs and symptoms of local nerve involvement arise, as they could signify potential malignancy-related local invasiveness. The most important of these signs are dysphagia and hoarseness [9, 16].

**Table 1:** Key questions for TN and thyroid gland patients Table content adapted from Tamhane et al. [9]

Questions
Do you experience problems swallowing?
Do you have breathing difficulties?
Have you noticed a change in your voice (hoarseness)?
Have you been coughing lately?
Have you noticed pain in any area of the neck?
Have you noticed anything strange or new within your neck?
Have you noticed a change in your weight?
Have you noticed that your hands are shaking?
Have you experienced any unusual sweating?
Have you or any of your relatives noticed some kind of change in your temper lately?
Are you more nervous than usual?

Special emphasis should be given to any exposure to childhood head/neck radiation, total body radiation due to bone marrow transplantation, fallout ionizing radiation, and/or significant neck mass growth. It is also vital to exercise vigilance towards any family history of thyroid cancer or associated syndromes such as multiple endocrine neoplasia type 2, familial adenomatous polyposis, or Cowden's syndrome [9].

After reviewing a patient's medical history, the evaluation progresses to a physical examination. This examination consists of two methods - inspection and palpation. The focus of an in-depth physical examination should be on assessing the thyroid gland's size, shape, and consistency. Additionally, any nodules present should be evaluated for location, number, size, solidity, tenderness, fixation to the surrounding structures, and the presence of other cervical masses, which can be metastases or lymphadenopathy. The eyes and orbit should also be assessed for signs of hyperfunctioning nodules such as exophthalmos, lid lag, and lid retraction [9]. Examining the thyroid is simplified if the patient has a long, slim neck. Nonetheless, if the patient has a short or thick neck or has had prior neck surgery, the examination may prove more difficult. This type of examination can occasionally be imprecise, and the accuracy of the thyroid palpation largely relies on the examiner's experience [7].

Diagnostic studies

An effective diagnostic framework is crucial for the proper management of patients with thyroid gland issues. Numerous diagnostic strategies have been devised to help create a streamlined approach to assessing TNs, as well as to bolster physicians' and patients' comprehension of the diagnosis and treatment of TNs. These methods should adhere to the clinical practice guidelines set forth by the American Association of Clinical Endocrinologists (AACE) and the American College of Endocrinology (ACE) [17].

Laboratory tests

Several serological tests can be employed to identify and analyze TNs.

Serum TSH

Patients with TNs should measure their serum TSH level to ascertain their thyroid's current functionality. This measurement will guide the subsequent medical examination. Also, if the TSH level proves abnormal, tests should be conducted for the patient's free T4 and free T3. A low TSH level suggests hyperthyroidism, either obvious or underlying. Therefore, an I-123 or pertechnetate scintigraphy scan should be the next step in assessing a patient with a low TSH level to examine the possibility of an autonomously functioning nodule. If the nodule is "hot", malignancy is unlikely, so an FNA can be postponed. If the serum TSH level exceeds the normal range or even hovers near its upper limit, this corresponds to an increased risk of malignancy, thus necessitating an FNA. Moreover, patients with heightened serum TSH levels should undergo hypothyroidism evaluations and have their thyroid antibodies measured [18].

Serum Calcitonin

Calcitonin levels in the serum can serve as an early detection marker for C-cell hyperplasia and medullary thyroid cancer (MTC). Patients with a family history or high probability of MTC or multiple endocrine neoplasia type 2 (MEN2) syndromes should have their calcitonin level checked. Screening with calcitonin has proven to be a practical and cost-efficient initial approach to assessing TNs [19]. Concerning calcitonin screening, it is crucial to recognize that both false-positive and false-negative outcomes might occur in patients with hypercalcemia, hypergastrinemia, neuroendocrine tumors, renal insufficiency, thyroid carcinomas, goiter, chronic autoimmune thyroiditis, and long-term usage of certain drugs [20]. Additionally, rare MTCs that do not secrete calcitonin might also yield false-negative results [21]. While many countries now include serum calcitonin level analysis as a standard part of evaluating patients with TNs, literature consensus on its recommendation remains unclear [1].

Serum Thyroglobulin (Tg)

The serum thyroglobulin level test is not typically performed during patient evaluations for TNs due to its lack of specificity and sensitivity in ruling out thyroid cancer. The usefulness of thyroglobulin levels in initial evaluations of TNs is limited because patients with thyroid disorders (especially multinodular diseases) often have similar thyroglobulin levels to those with thyroid cancer. However, this test can be useful when dealing with a patient who presents with a metastatic disease of unknown origin [22].

Imaging studies

Ultrasound Examination

Ultrasound (US) emerged as the preferred imaging tool for identifying and characterizing TNs due to the limitations of physical examinations. It is necessary for all patients with established or presumed TNs to undergo a thyroid ultrasound, as its findings can guide further investigation and management strategies. US is also used to analyze the composition, echogenicity, margins, calcification level, shape, and blood supply of TNs, as well as neighboring structures in the neck, including lymph nodes [8].

Ultrasonographic (US) features of TNs can indicate malignancy. Notably, some US characteristics within a TN can heighten the odds of distinguishing between benign and potentially malignant lesions. These include an unusual vertical shape measuring more in height than width, irregular margins, low echogenicity, microcalcifications, and lack of a halo [23]. Out of these, a TN that is taller than wide has been recognized as the most significant diagnostic factor in identifying malignancy [24].

Several US characteristics have been identified as strong predictors of benign nodules in the US. These include a purely cystic nodule, which has less than a 2% risk of malignancy [25], and a spongiform appearance, which is 99.7% likely to signify a benign TN [24]. Though ultrasound (US) exhibits considerable specificity in identifying suspicious features, it cannot wholly confirm a diagnosis or accurately differentiate between benign and malignant masses. However, recognizing at least two identified features can increase the likelihood of correctly identifying lesions that carry a high risk of malignancy [26].

Several US systems have been established to categorize the risk of malignancy in TNs and to standardize the terminology used by radiologists and endocrinologists. Table 2 provides a handy, three-tiered classification of malignancy risk: low, intermediate, and high, based on the ultrasound characteristics of TNs [16].

**Table 2:** Three-Tier Classification of the Risk of Malignancy Classification based on the Recent AACE Guidelines [16] FNA - fine-needle aspiration

Classes	Ultrasound features	Risk of malignancy	When to perform FNA
Class 1 (low risk)	Pure cyst or >50% cystic; isoechioic spongiform.	1%	>2.0 cm and larger
Class 2 (intermediate risk)	Slightly hypoechoic or isoechoic; ovoid with smooth or ill-defined margins; macrocalcifications or continuous rim calcification; intranodular vascularity	5-15%	>2.0 cm
Class 3 (high risk)	Marked hypoechogenicity; spiculated; microcalcification; taller-than-wide shape; evidence of extrathyroidal growth	50-90%	>1.0 cm

Another classification commonly used by medical professionals is the American College of Radiology Thyroid Imaging Reporting and Data System (ACR TI-RADS). This system has undergone multiple revisions, with the latest update released in 2017. TI-RADS proves valuable in categorizing nodules, using scores (TR1 to TR5) based on various features like composition, echogenicity, shape, margins, and echogenic foci. Each feature is assigned a score from 0 to 3 points. The total points determine the risk of malignancy according to five grades, with the grades corresponding to benign, minimally suspicious, moderately suspicious, or highly suspicious for malignancy. 

The TI-RADS scoring system offers a reliable framework for differentiating between benign and malignant nodules. Higher scores are more suggestive of malignancy, while lower scores (TR1) indicate a benign disease. Depending on the size of the nodule (15 mm or greater), the category TR3 is frequently regarded as a cutoff point for starting a follow-up.

The decision to perform fine-needle aspiration (FNA) or undergo ultrasound (US) follow-up is guided by the ACR-TI-RADS level and the maximum diameter of nodules. For risk grades TR3-TR5, a specified size threshold is identified for FNA. Additionally, lower size limits for follow-up US are established for TR3, TR4, and TR5 nodules, aiming to minimize repeat examinations for nodules likely to be benign or clinically insignificant. Highly suspicious nodules are subjected to biopsy only if they measure 1 cm or larger, while nodules with a low risk for malignancy should be further investigated only when their size reaches 2.5 cm or more.

Radionuclide Thyroid Scan/Scintigraphy

A radionuclide thyroid scan, common in nuclear medicine, uses iodine radioisotopes (usually 123I) or pertechnetate technetium (99Tc). This test measures the rate at which the thyroid gland absorbs these radioisotopes over time. Scintigraphy is used based on the understanding that malignant thyroid tissue typically does not process iodine. TNs are categorized according to the radionuclide uptake pattern. A "hot" TN is when the tracer uptake of radioisotopes exceeds that of the adjacent normal thyroid tissue, as seen in a hyper-functioning nodule [27]. Conversely, a "cold" TN is identified if there's a reduction or lack of tracer uptake, as in a hypo-functioning or non-functioning nodule. The standard uptake of radioisotopes is termed "warm" [28]. Hot nodules are seldom linked with malignancy, while warm nodules have an intermediate malignancy risk of approximately 5%. Cold nodules pose a 5-15% malignancy risk, even though more than 80% of TNs show benign pathology [1].

Computed Tomography and Magnetic Resonance Imaging 

Both CT and MRI offer limited usefulness in the early detection and evaluation of thyroid cancer within the thyroid gland. However, these tools are more valuable for pre-surgical assessments, especially for determining the extent of cancer spreading beyond the thyroid and regional metastasis.

Use of iodinated contrast-enhanced CT before surgery is discouraged as it could affect subsequent iodine-131 (I-131) uptake for post-operative thyroid ablation. Consider the necessary time gap between the iodinated contrast-enhanced CT and diagnostic imaging or radionuclide ablation if it has been used. The resumption of anti-thyroid drugs should occur only after the full effect of I-131, typically around six weeks. If concerns about incomplete clearance arise, urine iodine sampling can be conducted.

Accordingly, it is recommended to use non-contrast CT or contrast-enhanced MRI before surgery. Among these two, MRI offers superior soft tissue resolution. Additionally, contrast-enhanced MRI, using gadolinium, does not interfere with iodine uptake, unlike contrast-enhanced CT [29].

Single-Photon Emission Computed Tomography

Single-photon emission computed tomography (SPECT) is a technique in nuclear medicine that uses gamma rays to create tomographic imaging. Though it shares similarities with traditional nuclear medicine's planar imaging, which also employs a gamma camera, SPECT has the added benefit of producing true three-dimensional (3D) data. This tends to be displayed as cross-sectional patient slices that can be reformatted as needed. Since SPECT parallels planar gamma imaging, identical radiopharmaceuticals can be utilized. To gather SPECT images, patient projections are captured at set rotation points, typically every three to six degrees. For the best reconstruction, a full 360-degree rotation is used.

Each projection typically takes between 15 to 20 seconds, leading to a total scan time of approximately 15 to 20 minutes. SPECT imaging using technetium Tc 99m sestamibi (MIBI SPECT) is reported to have a 95% sensitivity and 100% specificity during parathyroid gland evaluations. It has also accurately identified malignant TNs in four out of five patients diagnosed with concurrent multinodular goiter.

Dual-isotope SPECT, using 99mTc-tetrofosmin and 123I sodium iodide, provides valuable diagnostic data for primary hyperparathyroidism patients. This method can conserve both operation time and costs. However, the pre-operative effectiveness of dual-isotope SPECT for tertiary hyperparathyroidism remains unclear. The application of newer PET tracers could potentially rectify this lack of diagnostic sensitivity.

Pre-operative SPECT imaging is employed in the evaluation of parathyroid adenomas in patients with multinodular goiters. This aids in identifying those who are appropriate candidates for minimally invasive radio-guided surgery. The technique also provides valuable insights into TNs that carry a suspicion of malignancy. The use of an intraoperative gamma-probe allows the surgeon to narrow their focus, receive real-time feedback on the operation's progression, minimize surgical trauma and complications, and achieve superior cosmetic outcomes. Patients with elevated pre-surgical parathyroid hormone levels may find particular benefit from radio-guided surgery [30].

Fine-Needle Aspiration (FNA)

FNA is recognized as the most precise test for diagnosing TNs. The introduction of this biopsy technique has significantly decreased the rate of unnecessary thyroid surgeries, boosted the detection of thyroid cancer in removed thyroid lobes, and helped save medical costs by identifying patients in need of surgical treatment. The fine-needle aspiration biopsie (FNAB) procedure is typically carried out in an outpatient setting and requires either no anesthesia or local anesthesia. It involves the use of a small-bore needle (23-27 gauge) to extract tissue samples for cytological analysis [31]. There are two possible techniques to guide the needle into the nodule using ultrasound.

The first, known as the in-plane approach, uses a parallel view where the needle orientation is along the transducer's long axis. The second method, the out-of-plane approach, involves a perpendicular view with the needle aligned to the transducer's short axis. The selection of either approach depends on the nature of the patient's lesion and overall condition. Some lesions may be more suitable for one method than the other [9].

Traditionally, FNA has been typically conducted using the manual palpation method. The size criteria for palpating TNs varies from 1.5 to 2.0 cm. As much as 30% of FNABs conducted without ultrasound (US) guidance can result in non-diagnostic outcomes. Occasionally, it is necessary to perform US-guided FNA (USGFNA) for nodules that are clinically non-palpable or technically challenging to aspirate using only palpation. An example of this would be predominantly cystic or posteriorly located nodules [1]. Higher-frequency transducers, between 7.5 and 10 MHz, can produce clearer images and offer continuous, real-time visualization of the needle tip, ensuring more precise sampling [32].

The diagnostic capabilities of FNA are regarded as top-notch. With an impressive diagnostic accuracy of 95%, a sensitivity scale between 68-98%, and a specificity scale between 72-100%, it stands out as a reliable procedure. Additionally, its false-negative rate is under 5%, and its false-positive rate is a low 1-3%. However, the accuracy and utility of FNA biopsies depend on the proficiency of both the individual conducting the biopsy and the cytologist, along with the criteria set for deeming a specimen adequate [33]. The results of the biopsy are categorized as either satisfactory or unsatisfactory (non-diagnostic) [16]. As per the Bethesda System for Reporting Thyroid Cytopathology, FNA results are interpreted as non-diagnostic, benign, indeterminate (atypia of undetermined significance/follicular lesion of undetermined significance (AUS/FLUS)), follicular neoplasm/suspicious for follicular neoplasm (SFN), suspicious for malignancy, or explicitly malignant. Every diagnostic group has a specific associated risk of malignancy, as detailed in Table 3 [34].

**Table 3:** The Bethesda System for Reporting Thyroid Cytopathology Table content adapted from Cibas et al. [34] AUS - atypia of undetermined significance; FLUS - follicular lesion of undetermined significance; FN - follicular neoplasm; SFN - suspicious for a follicular neoplasm

Diagnostic groups	Bethesda category	predicted risk of malignancy
Inadequate/non-diagnostic	I	1-4%
Benign	II	0-3%
Follicular lesion of undetermined significance (AUS/FLUS)	III	5-15%
Neoplasm FN/SFN (follicular or oncocytic)	IV	15-30%
Suspicious for malignancy	V	60-75%
Malignant	VI	97-99%

The FNA procedure has certain limitations. Its outcome can be non-diagnostic, producing suspicious or indeterminate results. Non-diagnostic FNA results often originate from aspirates with insufficient cells for diagnosis or errors caused by incorrect needle positioning. In such instances, the procedure should be repeated using ultrasound guidance. The Bethesda System, which provides guidelines for reporting thyroid fine-needle aspirations (FNAs), suggests that repeat aspirations should be conducted at a minimum of three months after the initial aspiration. This practice aims to avoid misinterpretations of false-positive results caused by reactive or reparative changes. Suspicious or indeterminate FNA results usually stem from the challenges of differentiating benign Hurthle cell and follicular cell neoplasms from their malignant equivalents based solely on cytology. As a result, surgical removal of follicular neoplasms is typically suggested in these scenarios [9].

Update on the evaluation of thyroid nodules

Diffusion-Weighted Magnetic Resonance Imaging

Originally utilized for neuro-imaging, diffusion-weighted (DW) MRI now services a broad array of extracranial applications. These include differentiating benign from malignant nodules, aiding in tumor staging, and identifying post-operative recurrence and residual tumors in the head and neck regions. Furthermore, DW MRI can assess therapeutic responses in these tumors and differentiate between recurring and post-therapeutic changes at the surgery site [35].

The advancement of echo-planar imaging (EPI) has broadened the scope of diffusion-weighted imaging (DWI), allowing for superior image quality with fewer motion-related errors. Notably, applying DWI to TNs offers valuable insight into their characteristics and assists in distinguishing between benign and malignant nodules. However, there are limited studies to date that have investigated the use of DWI in determining the malignancy of TNs [36].

Magnetic Resonance Spectroscopy (MRS)

Researchers have investigated various magnetic resonance (MR) techniques, including spectroscopy (MRS), to distinguish between benign and malignant thyroid tissue. Initially, these techniques were employed to identify the morphological characteristics of cancers. Now, they can detect the biochemical differences between benign or malignant thyroid neoplasms and normal thyroid tissue. The effectiveness of these techniques may be evaluated in a subsequent phase, which entails differentiating between benign and malignant neoplasms. Previous studies typically used samples from surgically removed thyroids, and the findings were positive, contributing to pre-operative preparations for thyroid neoplasm surgeries [37]. Notably, a pre-clinical version of MRS was tested on samples obtained via FNA. Concerns were also raised about whether this technique could notably improve the pre-operative diagnosis of indeterminate thyroid lesions [37].

Optical Coherence Tomography and Optical Coherence Microscopy

Optical coherence tomography (OCT) and optical coherence microscopy (OCM) are emerging techniques that utilize intrinsic optical contrast for imaging. OCT provides high-quality, real-time, cross-sectional images of tissues, while OCM is an advanced version of OCT that magnifies cell images considerably (Figure 4). The OCT/OCM system employs infrared light to imitate gland microstructures within a cell range of 1-15 μm. This process generates high-resolution images that surpass those produced via histopathological techniques. Furthermore, OCT and OCM can effectively distinguish between healthy and cancerous TNs through intrinsic visual contrast [38].

**Figure 1:**
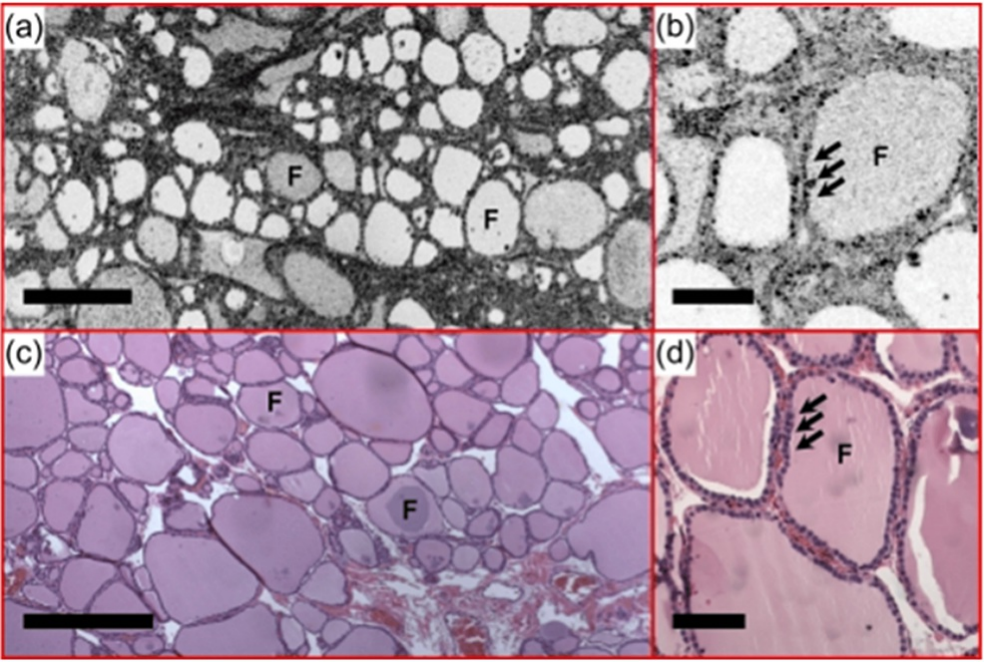
Ultrahigh resolution optical coherence tomography Normal thyroid demonstrates well-organized round to oval thyroid follicles (f). Colloid with various densities was observed under (a) en face OCT and (b) OCM. The follicles are lined by a single layer of epithelium (arrows), which are clearly seen in the OCM image (b); c and d are corresponding H&E slides. OCT - optical coherence tomography; OCM - optical coherence microscopy Permission to use the figure in this review article has been obtained from Society of Photo-optical Instrumentation Engineers, International Biomedical Optics Society, Journal of Biomedical Optics [38].

Molecular Markers

Molecular markers in TNs are advised for use in cases where cytological diagnosis is uncertain to assist in surgical decision-making. Needle-washing samples are collected during FNAB, with subsequent molecular tests producing the majority of data.

Afirma Gene Expression Classifier

The Afirma gene expression classifier (GEC), which measures the mRNA expression of 167 genes, is utilized to detect benign gene expression profiles. Though its sensitivity and negative predictive value stand high at 92% and 93%, respectively, it has disappointingly lower positive predictive value and specificity (48-53%). The GEC works as a screening test to rule out benign TNs. A benign GEC result implies a minimal risk of malignancy; it should be noted that this does not completely eliminate the possibility of cancer, which still stands at a 5% risk [39].

A Seven-Gene Panel of Genetic Mutations and Rearrangements

The seven-gene mutation and rearrangement analysis panel evaluate BRAF, NRAS, HRAS, and KRAS point mutations, along with the common rearrangements of RET/papillary thyroid cancer (PTC) and PAX8/PPARγ. It boasts a high specificity of 86-100% [40], including a positive predictive value of 84-100%, although it falls short when it comes to sensitivity, with rates ranging from 44-100%. This analysis panel is currently employed in the diagnosis of thyroid malignancy. Molecular testing is a rapidly evolving field, and many more tests, including mRNA and miRNA markers, are under development [41]. Consequently, patients should be educated about the benefits and limitations of molecular testing. Currently, the AACE guidelines neither promote nor discourage the use of molecular tests in clinical practice [16]. However, this stance may change as further advancements are made in molecular test development [9].

Management and follow-up

Several factors should be taken into account for the management of TNs. These include serum TSH level, assessment of clinical risk factors, nodule size, ultrasound features, patient preferences, and FNAB results. Among these, the FNAB-based cytological diagnosis is arguably the key determinant in decision-making.

Below, we outline the practical management of TNs after FNA, the associated malignancy risks, and recommended management strategies [42].

The practical management of thyroid nodules after FNA

Nodules Associated With Hyperthyroidism 

There are several management options for patients with hyperthyroidism. These include stabilization using anti-thyroid medications, followed by either radioiodine therapy or surgery, unless these options are absolutely contraindicated. Patients diagnosed with subclinical hyperthyroidism, characterized by low TSH and normal FT4, present a certain controversy regarding their treatment criteria. The need for treatment depends on the clinical risk of complications linked to its potential negative impact on the heart and bone. This is especially important in elderly patients over 60 years old who have atrial fibrillation and postmenopausal women at risk of osteoporosis. The degree of TSH suppression should also be considered when deciding treatment protocols [42].

Incidentaloma

An incidentaloma commonly refers to an undetectable, symptomless TN, which could be benign or malignant, discovered through imaging. It carries the same malignancy risk as a noticeable nodule. Generally, nodules accidentally detected under 1cm in size should not be biopsied and require careful follow-up [1].

Nodules Selected for FNA

The diagnostic categories from Bethesda, their respective malignancy risks, and recommended management options are outlined below (Table 4) [43].

**Table 4:** The frequency and duration of the follow-up of benign TNs Table content adapted from Haugen et al. [1] US - ultrasound; FNA - fine-needle aspiration

Nodule's features	Duration of follow-up
Nodules with high suspicious US pattern	Repeat US and FNA within 12 months
Low to intermediate suspicious US pattern	Repeat US in 12-24 months.
Nodules with very low suspicious patterns	US repeated at 24 months or more.

Category I - non-diagnostic: Non-diagnostic biopsies (Bethesda I) are often linked to cytologically inadequate samples. However, the lack of malignant cells does not necessarily mean a negative biopsy. If a patient's initial FNAB yields non-diagnostic results, Ultrasonography (US)-guided FNA should be conducted in a four- to six-week time frame. Most clinicians commonly recommend waiting around three months before retesting, giving any inflammation from the initial FNA time to subside.

If the specimens are inconclusive, a core needle biopsy, careful monitoring (especially if the nodule is solid), or diagnostic surgical excision may be considered. This decision would be based on the ultrasound pattern, the presence of clinical risk factors, and the detection of rapid nodule growth [1].

Category II - benign nodules: Patients with benign nodules (Bethesda II) receive synthetic thyroxine to control their TSH levels. These patients then have a clinical and ultrasonography follow-up between 6 and 18 months, assuming the nodule is not palpable. Continuous, cautious monitoring of any changes in the nodule size or related symptoms is highly recommended [34].

If the TN enlarges, follow-up actions such as repeated ultrasonography or FNAB should be undertaken. However, the regularity and duration of monitoring benign TNs can vary in clinical practice (Table 4). Significant growth in the nodule, manifesting as an increase of more than 50% in its volume or a less than 20% increase in at least two of its solid dimensions or within the solid section of cystic-solid nodules, necessitates its removal via thyroid lobectomy [1]. Surgical removal might be necessary for benign TNs if they cause pressure or structural symptoms such as difficulty swallowing, choking, voice changes, difficulty breathing, or pain [16].

Categories III and IV - indeterminate nodules (FLUS/AUS or FN/SFN): Indeterminate cytology, specifically Bethesda III and IV, presents a diagnostic challenge due to variations in cytopathological findings, which can hinder clear cytologic diagnosis [34].

The management methods for indeterminate TNs can vary greatly. A unilateral lobectomy, or hemithyroidectomy, is often the standard procedure for confirming a histopathological diagnosis if repeated FNABs or molecular analysis suggest malignancy. However, as only 10-40% of these cases turn out to be malignant, this carries a risk of unwarranted surgery. Approximately 60% of patients with indeterminate cytology results might have papillary thyroid carcinoma - a type of cancer [44].

If benign follicular adenoma is indicated from surgical histology results, the contralateral thyroid lobe usually suffices for maintaining euthyroidism, and thyroxine treatment is not necessary. However, if cancer is indicated, a complete thyroidectomy is recommended to reduce the risk of hypoparathyroidism and recurrent laryngeal nerve injury. Particularly, in special cases like bilateral lesions or significant enlargement of the contralateral lobe, a total or near-total thyroidectomy can be justified [45]. Molecular analysis, a technique with documented positive predictive values, can be beneficial for indeterminate FNAB results. However, due to high costs, many institutions still lack access to genetic testing [46].

Category V - suspicious for malignancy: Specimens strongly suggestive of PTC but lacking diagnostic evidence are called suspicious cytology (Bethesda V) [34]. Patients with a TN and an FNAB indicating possible PTC should undergo surgery, as should patients with confirmed malignant cytology [1]. Consider utilizing molecular markers for definitive total thyroidectomy decision-making only if there's no other indication for the procedure and if it significantly impacts surgical decisions [1].

Category VI - malignancy: Malignant cytology (Bethesda VI) signifies samples satisfying all malignancy criteria, thus confirming a cancer diagnosis [34]. This category comprises papillary and follicular carcinomas, Hurthle cell (oncocytic) carcinoma, medullary cancer, thyroid lymphoma, anaplastic cancer, and cancer that metastasizes to the thyroid [1,6]. Generally, patients diagnosed with malignant cytology should be referred to a seasoned thyroid surgeon for a complete thyroidectomy. Post-operative care should then be managed by an endocrinologist [9].

**Figure 2:**
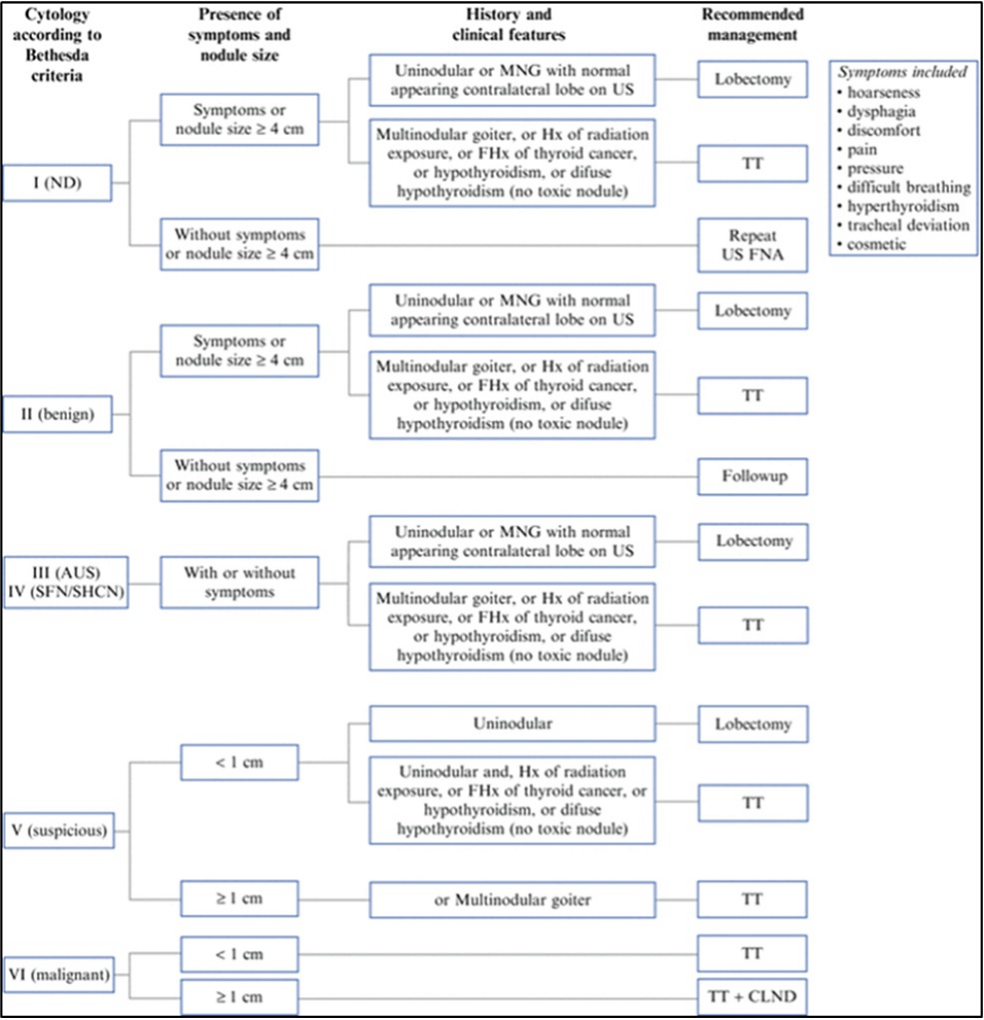
Treatment approach to thyroid nodule Permission to use the figure in this review article has been obtained from Annals of Surgical Oncology, Springer Nature [43] ND - non-diagnostic; TT - thyroxine treatment; US FNA - ultrasound fine-needle aspiration; AUS - atypia of undetermined significance; SFN/SHCN - suspicious for follicular neoplasm/ suspicious for Hurthle cell neoplasm; CLND - central lymph node dissection

Treatment approaches to TN are shown in Figure 2.

Alternative treatments/ or non-surgical treatments of the thyroid nodules

Several non-surgical methods are available for treating and monitoring TNs.

Radioactive Iodine

Radioactive iodine is primarily used to treat hyperthyroidism, a condition of an overactive thyroid. Upon treatment, it gets absorbed by the thyroid, which often results in reduced nodules and a decrease in hyperthyroidism symptoms, typically within 2 to 3 months. This treatment significantly reduces the thyroid gland's thyroxine production and may also decrease gland size. It is not suitable for pregnant women or those trying to conceive [5].

Anti-Thyroid Medications

In certain situations, the anti-thyroid drug methimazole (Tapazole) could be prescribed to alleviate hyperthyroidism symptoms. This treatment, typically long-term, could potentially pose serious risks to the liver [5].

Thyroid Hormone Treatment

Thyroid hormone treatment can replace the function of a malfunctioning thyroid gland. Moreover, it can serve as a suppression therapy to inhibit additional thyroid tissue growth. This therapy is primarily used in thyroid cancer patients to prevent cancer recurrence or progression. The safety and effectiveness of levothyroxine (LT4) suppressive therapy remain a topic of debate. As such, this treatment is not widely recommended [5].

Radiofrequency Ablation (RFA) and Percutaneous Ethanol (EtOH) Injection

Radiofrequency ablation (RFA) and percutaneous ethanol (EtOH) injection are contemporary minimally invasive alternatives for managing local or regional recurrence of well-differentiated thyroid cancer (WTC) instead of surgical intervention. Both techniques have their own advantages and disadvantages. RFA offers a broader zone of tumor destruction with precise energy adjustment, making it suitable for treating larger lesions. However, the heightened energy increases the risk of causing permanent injury to adjacent nerves. To mitigate this risk, a protective thermal barrier in the form of a 5% dextrose in water solution can be injected between the mass and the anticipated location of the nerve during RFA. RFA is generally favored for lesions exceeding 10 mm and those located outside the neck, while EtOH is preferred for neck lesions under 10 mm or those closely situated to nerves. The choice between RFA and EtOH as the primary ablative therapy depends on the size and anatomical location of the lesion. In RFA treatment, concern should prioritize avoiding thermal damage to nerves over blood vessels, as the circulating blood within vessels efficiently dissipates heat from the RF applicator. Special care is warranted when treating lesions within the lateral aspect of the central compartment, particularly those in close proximity to the recurrent laryngeal nerve [47].

Thyroid Artery Embolization 

Thyroid artery embolization (TAE) can significantly reduce the volumes of sizable thyroid nodules in a three-month follow-up, leading to notable improvement in both quality of life and clinical symptoms. The procedures were well tolerated by patients and did not show any significant complications post-TAE. Consequently, TAE stands as a viable alternative therapy to surgery or percutaneous ablation, particularly in selected cases with large thyroid nodules [48]. 

## Conclusions

This review discusses the current understanding of TNs, their properties, and how to effectively manage them. The main challenge lies in striking a balance between excessive investigation and treatment, alongside the risk of under-treating potentially significant thyroid cancer. It is essential to use logical, evidence-based practice to avoid unnecessary testing, procedures, and surgeries that could induce stress and negatively affect patients. Taking into account the risk factors, initial clinical and laboratory evaluations of TNs guide the selection of patients who require further investigations to rule out cancer. Key factors of ultrasound examinations include identifying nodule features that may indicate malignancy and facilitating decision-making about which TNs need more invasive procedures and which can be monitored without intervention, as per risk stratification systems. FNA is deemed the most effective technique to sample TNs and help identify which require surgery and which do not. Treatment of TNs varies from low-risk follow-ups to high-risk surgical interventions. It is essential to assess each patient individually, using a multidisciplinary approach. The best approach for each TN patient can be determined post-risk evaluation, following current guidelines.

This review suggests that future work should focus on developing a more efficient testing algorithm. It should have a high negative predictive value for cytologic samples of indeterminate significance. This will enhance confidence in opting for a treatment approach of watchful waiting or inaction.
